# Editorial: Model organisms in experimental pharmacology and drug discovery 2023: rodent, worm and zebrafish models

**DOI:** 10.3389/fphar.2024.1462972

**Published:** 2024-08-08

**Authors:** Kuo-Hui Su, Priscilla Kolibea Mante, Pedro Xavier-Elsas

**Affiliations:** ^1^ Department of Cell and Cancer Biology, College of Medicine and Life Sciences, The University of Toledo, Toledo, OH, United States; ^2^ Department of Pharmacology, Kwame Nkrumah University of Science and Technology, Kumasi, Ghana; ^3^ Department of Immunology, Federal University of Rio de Janeiro, Rio de Janeiro, Brazil

**Keywords:** model organisms, drug discovery, pharmacology, neuroprotection, neuromuscular, pulmonary hypertension, fermented teas, pigmentation disorders

Model organisms, such as rodents, worms, and zebrafish, have become increasingly essential in the biomedical research field (Konno et al., 2020). We compiled two research articles and three review articles for the Research Topic “*Model organisms in experimental pharmacology and drug discovery 2023: rodent, worm and zebrafish models*” Each article offers unique contributions to the broader landscape of experimental pharmacology and drug discovery, showcasing diverse methodologies and innovative approaches.

Parkinson’s disease (PD) is a neurodegenerative disorder that leads to the progressive degeneration of dopamine-producing neurons in the brain (Dexter and Jenner, 2013). Finding highly effective neuroprotective treatments for PD is imperative. Zebrafish as model organisms have demonstrated immense value for researchers investigating disease causes and potential therapeutics owing to their genetic and physiological resemblances to humans (Razali et al., 2021). Omar et al. investigated the effects of neurotrophin-3 (NT3), a neuroprotective growth factor, on the protection of dopaminergic neurons in a zebrafish PD model. They found NT3 mRNA to have widespread expression in neurons across the whole zebrafish brain. Exposure to neurotoxins leads to a significant decrease in movement, reduced expression of dopamine-related genes, and reduced number of dopaminergic neurons in the brain. The administration of recombinant NT3 (rNT3) demonstrated significant improvements in locomotor function and an observed increase in the number of dopaminergic neurons. From a biochemical perspective, rNT3 decreases caspase-3 levels and increases levels of glutathione S-transferase, indicating a reduction in oxidative stress and apoptosis. This study highlights the possibility of NT3 protecting the brain from PD and rNT3 being a potential therapy for neurotoxicity. It provides important information about how NT3 promotes neuronal survival, regulates dopamine, and enhances overall brain health, as demonstrated in the zebrafish model.

Another research article uncovered the role of carveol, a type of monoterpene present in spearmint essential oil, in the neuromuscular system. The increasing resistance of parasitic nematodes to conventional anthelmintics highlights the pressing necessity to explore novel pharmacological agents (Hahnel et al., 2020). Monoterpenes found in essential oils have been identified as promising candidates due to their bioactive characteristics. Stojković et al. proposed using carveol as a substitute anthelmintic agent to combat the increasing resistance to current medicines. The study employed multiple models, including the free-living nematode *Caenorhabditis elegans*, the parasitic nematode *Ascaris suum*, and mammalian tissues (rat ileum and diaphragm) to assess the pharmacological effects of carveol. Carveol demonstrated a strong binding affinity for the orthosteric location of the nicotinic acetylcholine receptor (nAChR) in *Ascaris suum*. Carveol induced spastic paralysis and markedly decreased pharyngeal pumping in *Caenorhabditis elegans*. In the *A. suum* model, carveol potentiated the effects of acetylcholine (ACh)-induced contractions by decreasing the Half maximal effective concentration and raising the maximum response. Some of these effects were responsive to atropine, suggesting the involvement of both nicotinic and muscarinic receptors. Carveol potentiated the effectiveness of ACh in *Xenopus laevis* oocytes that expressed *A. suum* nAChR. Additionally, it functioned as a noncompetitive antagonist of ACh in mammalian models, decreasing the strength of contractions in the rat ileum without altering the EC50 value. Carveol exhibited a biphasic effect on contraction in the rat diaphragm, enhancing it at lower concentrations and inhibiting it at higher ones. This indicates an intricate interplay with nicotinic receptors. This research reveals that carveol exhibits potential for the development of novel anthelmintics and offers valuable insights into the mechanics of neuromuscular neurotransmission.

Zebrafish are recognized for their genetic similarities to humans and transparent embryos, making them ideal for studying skin pigmentation disorders (Russo et al., 2022). Qu et al. detailed the structure of zebrafish skin and its melanin production pathways, which are closely aligned with those in humans. This review discusses the effectiveness and limitations of methods for creating zebrafish models of abnormal skin pigmentation, including physical (ultraviolet radiation and pulsed electromagnetic fields), chemical (such as 5-HT, fisetin, flumequine, and heterocyclic organic compound protoporphyrin IX), and genetic approaches. This review highlights the critical role of zebrafish in advancing dermatological research and the significant impact of pigmentation disorders, which range from cosmetic issues to severe hyperpigmentation diseases affecting psychological and economic wellbeing.

There is a critical need for effective therapeutic strategies targeting pulmonary hypertension (PH) associated with left heart disease (PH-LHD) (Ltaief et al., 2023). Jasińska-Stroschein examined systemic hypertensive models, pressure-overload-induced heart failure (HF), ischemic heart failure, and high-fat diet metabolic models to investigate and mimic PH-LHD rodent models, comparing their effectiveness in mimicking human disease conditions. This review quantitatively compares the efficacy of popular approaches to address PH-LHD based on numerous experimental protocols examining the phenotypical characteristics of both PH and left ventricle performance. It analyzes the details of heart failure models that can promote pulmonary hypertension, the extent of this promotion, and the primary determinants of severity in PH-LHD models. The findings could enhance the rigor and quality of preclinical studies.

Epidemiological studies, animal experiments, and cell experiments reveal that fermented teas are beneficial in regulating bone mass (Shen et al., 2009). Xu et al. summarized experimental studies in rodent models that have shown the positive effects of fermented tea on bone health, such as improving bone density and microstructure, enhancing calcium absorption, and reducing markers of bone resorption. The major active components in fermented tea are tea polyphenols, tea pigments, and trace amino acids. These components work through several mechanisms: they regulate bone marrow mesenchymal stem cell osteogenesis, inhibit osteoclast activity, promote calcium and phosphorus absorption, reduce inflammation levels, regulate gut microbiota, modulate endocrine function, and inhibit oxidative stress. This review highlights the potential of fermented tea as a functional drink for osteoporosis prevention and bone mass regulation.

In summary, the research articles in this Research Topic reflect the multifaceted and dynamic character of experimental pharmacology and drug discovery. They serve as examples of how innovative research can result in substantial advancements in the comprehension and treatment of complex maladies. The insights gained from these studies will inform and inspire future research and drive progress in biomedical science and therapeutic development.
